# Primary breast diffuse large B‐cell lymphoma in the rituximab era: A retrospective study of the Chinese Southwest Oncology Group

**DOI:** 10.1002/cam4.6686

**Published:** 2023-11-23

**Authors:** Huawei Weng, Prem Raj Shrestha, Huangming Hong, Zegeng Chen, Le Yu, Yuyi Yao, Zhihui Zhang, Liqun Zou, Bo Zhu, Hui Zhou, Xianling Liu, Yao Liu, Hongqiang Guo, He Huang, Tongyu Lin

**Affiliations:** ^1^ Department of Medical Oncology Sun Yat‐sen University Cancer Center, State Key Laboratory of Oncology in South China, Collaborative Innovation Center for Cancer Medicine Guangzhou China; ^2^ Department of Medical Oncology Sichuan Cancer Hospital & Institute, Sichuan Cancer Center, Affiliated Cancer Hospital of University of Electronic Science and Technology of China Chengdu China; ^3^ Division of Medical Oncology, Cancer Center and State Key Laboratory of Biotherapy Sichuan University, West China Hospital Chengdu China; ^4^ Institute of Cancer, Xinqiao Hospital Army Medical University Chongqing China; ^5^ Tumour Hospital of Xiangya School of Medicine Central South University Changsha Hunan China; ^6^ Department of Oncology, Secondary Xiangya Hospital Central South University Changsha Hunan China; ^7^ Chongqing University Cancer Hospital Chongqing China; ^8^ The Affiliated Cancer Hospital of Zhengzhou University Henan Cancer Hospital Zhengzhou China

**Keywords:** CNS prophylactic treatment, genetic mutation characteristics, high‐dose methotrexate, primary breast diffuse large B‐cell lymphoma, radiotherapy

## Abstract

**Background:**

Primary breast diffuse large B‐cell lymphoma (PB‐DLBCL) is a rare subtype of extranodal DLBCL, and the standard treatment remains controversial. In this study, we aimed to define the optimal treatment management in the rituximab era.

**Methods:**

A total of 5089 newly diagnosed DLBCL patients treated with rituximab‐containing immunochemotherapy between 2008 and 2019 from the Chinese Southwest Oncology Group‐affiliated institutes were identified, of whom 135 diagnosed with PB‐DLBCL were eligible for this analysis.

**Results:**

PB‐DLBCL accounted for 2.7% of all DLBCLs. With a median follow‐up of 4.2 years, the 5‐year overall survival and progression‐free survival rates were 84.8% and 71.6%, respectively. Breast and central nervous system (CNS) relapses were the main cause of treatment failure. We observed that consolidative breast radiotherapy (RT) significantly decreased breast relapse risk (5‐year risk, 2.9% vs. 20.1%, *p* = 0.007). The CNS relapse risk was lower for patients who received high‐dose methotrexate (HD‐MTX) than for patients who did not (5‐year risk, 0% vs. 15.2%, *p* = 0.015). We further screened the genetic mutation profile of 20 patients from two institutes, and found that *MYD88* (25%) and *CD79B* mutations (25%) frequently occur in PB‐DLBCL. In addition, four patients with *MYD88* and/or *CD79B* mutations experienced CNS relapse, while three patients with *MYD88* and/or *CD79B* mutations who received HD‐MTX did not experience CNS relapse.

**Conclusion:**

Collectively, our results indicate combined modality therapy including rituximab‐containing immunochemotherapy and consolidative breast RT is a promising approach for PB‐DLBCL, while HD‐MTX is useful for preventing CNS relapse.

## Graphical abstract

This multicenter retrospective study presents a comprehensive picture of the clinical and genetic features of primary breast diffuse large B‐cell lymphoma (PB‐DLBCL) in the rituximab era. Our results showed that consolidative breast radiotherapy (RT) could significantly reduce the risk of breast relapse and ultimately lead to improved survival outcomes, while HD‐MTX reduced CNS relapse risk.

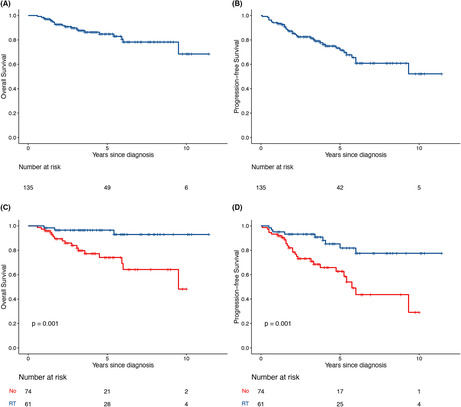

## 1 INTRODUCTION

Primary breast lymphoma (PBL) is a rare subtype of extranodal non‐Hodgkin lymphoma (NHL), accounting for an estimated 1% of NHL and 0.5% of all breast tumors.^1^, ^2^, ^3^ According to Wiseman and Liao,^4^ PBL is defined as primary lymphoma occurring in the breast with or without regional lymph node involvement and without previously diagnosed extramammary or concurrent disseminated disease. Histologically, diffuse large B‐cell lymphoma (DLBCL) is the most common subtype, comprising 56%–84% of cases.^5^, ^6^, ^7^, ^8^

The combination of rituximab plus cyclophosphamide, doxorubicin, vincristine, and prednisolone (R‐CHOP) has been established as the standard for the first‐line treatment of DLBCL patients in the modern era.^9^ However, patients with primary breast (PB)‐DLBCL exhibit distinct clinicopathological features compared to nodal or other extranodal DLBCL, which shows a tendency to relapse in the central nervous system (CNS) and breast.^5^, ^10^ There is a relative deficiency of data on relapse patterns and treatment efficacy in patients with PB‐DLBCL treated with rituximab‐containing immunochemotherapy in the modern era. It has been questioned whether PB‐DLBCL requires different treatment management. Due to the rarity of PB‐DLBCL, most available data have been derived from either small retrospective series^5^, ^8^, ^10^, ^11^, ^12^, ^13^, ^14^, ^15^, ^16^, ^17^ or nonrandomized phase 2 studies.^18^, ^19^, ^20^ Furthermore, CNS prophylaxis was minimally used in most retrospective series, making it difficult to assess the impact on CNS relapse.

Herein, we conducted a multicenter retrospective study to describe the clinical characteristics, survival outcomes, and pattern of relapses of PB‐DLBCL in the rituximab era, thus helping to define the optimal management of PB‐DLBCL. In addition, we tried to elucidate the genomic mutation profile and provide insights into the pathogenesis of PB‐DLBCL.

## 2 MATERIALS AND METHODS

### 2.1 Patients

We conducted a multicenter retrospective analysis of consecutive patients with DLBCL diagnosed between January 2008 and December 2019 from eight centers in the Chinese Southwest Oncology Group (CSWOG). Patients with a pathologic confirmation of PB‐DLBCL and those who received frontline rituximab‐containing immunochemotherapy were included. PB‐DLBCL was defined as DLBCL involving only one or both breasts with or without ipsilateral regional lymph node involvement. Patients with systemic disease with breast involvement, transformation of a previous indolent lymphoma, commitment to other cancers, or did not receive any treatment after diagnosis were excluded. All PB‐DLBCL cases included in the final analyses had undergone central pathology review by experienced hematopathologists, and all cases were reclassified according to the 5th edition of the World Health Organization classification of hematopoietic and lymphoid tumors.^21^ All patients were staged according to the Ann Arbor staging system. The stage of patients with bilateral breast involvement was determined by the degree of involvement of other nodal or extranodal sites.

The collected data included clinical characteristics, stage, laboratory data, stage‐modified International Prognosis Index (SM‐IPI) that is based on age >60 years, elevated lactated dehydrogenase (LDH), performance status ≥2 and stage II or IIE,^22^ pathologic information, initial treatments, relapses or progression of disease, and follow‐up examinations. The Hans algorithm was used to classify patients as germinal center B‐cell‐like phenotype (GCB) or nongerminal center B‐cell‐like (non‐GCB).^23^ Staging workup and initial treatment for patients were performed according to local clinician discretion. CNS prophylaxis was administered based on local clinician's preference. This study was approved by the Institutional Review Board.

### 2.2 Targeted sequencing

Targeted sequencing covering exons and selected introns of leukemia‐ and lymphoma‐related genes was peformed for 20 patients with PB‐DLBCL patients from two of these institutes. Formalin‐fixed, paraffin‐embedded tumor tissues were used for genomic DNA extraction with a QIAamp DNA FFPE Tissue Kit (Qiagen), and the paired normal control DNA of peripheral blood mononuclear cells (PBMCs) was extracted with a DNeasy Blood & Tissue kit (Qiagen) following the manufacturer's instructions. Tumor genomic DNA and matched PBMCs were fragmented into 300–350 bp fragments using a Covaris M220 instrument (Covaris). Sequencing libraries were prepared with a KAPA Hyper Prep kit (KAPA Biosystems) with optimized protocols. Libraries were then subjected to PCR amplification and purification before targeted enrichment. The probes for targeted sequencing panel covered exons and selected introns of leukemia‐ and lymphoma‐related genes and were produced by Nanjing Shihe Jiyin Biotechnology Inc. (Nanjing, China). Afterward, the samples were purified by Agencourt AMPure XP beads, quantified by a KAPA Library Quantification kit (KAPA Biosystems), and sized with an Agilent Technologies 2100 Bioanalyzer (Agilent Technologies). Finally, the enriched libraries were sequenced on HiSeq 4000 NGS platforms (Illumina) to coverage depths of 1500× after removing PCR duplicates for FFPE.

### 2.3 Statistical analysis

Patient characteristics were compared using the Mann–Whitney *U* test for continuous variables and the chi‐squared test or Fisher's exact test for categorical variables. Progression‐free survival (PFS) was defined as the time from initial diagnosis to disease progression or death from any cause. Overall survival (OS) was defined as the time from initial diagnosis to death from any cause. Time to specific site relapse was calculated from the date of diagnosis to the date of relapse. The median follow‐up time was estimated using a reverse Kaplan–Meier method.^24^ Survival curves were estimated using the Kaplan–Meier method, and comparisons between groups were calculated using the log‐rank test. Univariate and multivariate analyses for OS and PFS were performed using the Cox regression method. All the variables with *p* < 0.1 in univariate analysis were included in the multivariate analysis. A two‐tailed *p* < 0.05 was considered statistically significant. Statistical analyses were performed using SPSS version 26.0 (IBM, Armonk, NY, USA).

## 3 RESULTS

### 3.1 Patient characteristics

We identified 5089 newly diagnosed DLBCL patients treated with rituximab‐containing immunochemotherapy from 2008 to 2019 at eight CSWOG‐affiliated institutions, in 137 patients (2.7%) who were diagnosed with PB‐DLBCL. Two patients were excluded for previously diagnosed indolent lymphoma (*n* = 1) and commitment to other cancers. Finally, 135 patients were included in the analyses.

The baseline characteristics are summarized in Table 1. The median age at diagnosis was 51 years (range, 19–82 years). The bilateral breast involvement occurred in only 10 patients (5.2%) at presentation. Sixty‐five (48.1%) patients had stage IE disease, and 70 (51.9%) had stage IIE disease. By SM‐IPI, most patients (75.6%) were classified as low risk (0‐1) and 24.4% as high risk (2–3). Non‐GCB was the most common phenotype (*n* = 92, 68.1%) and dual expression was observed in 59 (43.7%) patients. Fluorescence in situ hybridization studies for *MYC* and *BCL2* and/or *BCL6* rearrangements were performed in 27 patients, and only one patient had double‐hit lymphoma. For staging workup, 84.4% of patients (*n* = 114) had PET/CT scans, and 25.6% (*n* = 21) had CT or MRI scans.

**TABLE 1:** Baseline characteristics.

Characteristic	All, *n* (%)
Number	135
Median age (range), years	51 (19–82)
≤60	103 (76.3)
>60	32 (23.7)
Gender	
Female	133 (98.5)
Male	2 (1.5)
ECOG	
0–1	134 (99.3)
≥2	1 (0.7)
B symptoms	
Present	8 (5.9)
Absent	127 (94.1)
Primary site	
Left	60 (44.4)
Right	65 (48.1)
Bilateral	10 (7.4)
Median tumor size (range), cm	3.2 (0.8–22.8)
Bulky disease (>7 cm)	12 (8.9)
Regional lymph node involvement	
Axillary	59 (43.7)
Supraclavicular ± axillary	11 (8.1)
None	65 (48.1)
Ann Arbor stage	
IE	65 (48.1)
IIE	70 (51.9)
Serum LDH	
Elevated	32 (23.7)
Normal	103 (76.3)
SM‐IPI	
0	40 (29.6)
1	62 (45.9)
2	27 (20.0)
3	6 (4.4)
Cell of origin	
Germinal center	43 (31.9)
Non‐germinal center	92 (68.1)
Dual expression	59 (43.7)

Abbreviations: ECOG, Eastern Cooperative Oncology Group; LDH, lactate dehydrogenase; SM‐IPI, stage modified International Prognostic Index.

### 3.2 Treatment and response

The first‐line treatment is summarized in Table 2. A total of 13 patients (9.6%) underwent surgical resection before treatment. Rituximab‐containing immunochemotherapy was given to all patients. In terms of chemotherapy regimens, 115 (85.2%) received CHOP, 11 (8.1%) received EPOCH, 5 (3.7%) received CHOEP, and 4 (3.0%) received Hyper CVAD. The median number of chemotherapy cycles overall was 6 (range, 1–8). Radiotherapy (RT) to the ipsilateral breast with or without regional lymph nodes was given in 61 patients (45.2%), with two patients also receiving prophylactic RT to the contralateral breast. The median cumulative dosage of RT was 36.0 Gy (range, 25.2–55.0 Gy). Sixty‐nine patients (51.1%) received CNS prophylaxis, including 34 patients who received intrathecal (IT) prophylaxis and 35 patients who received high‐dose methotrexate (HD‐MTX). Among patients who received HD‐MTX, 26 also received concomitant IT prophylaxis. HD‐MTX was intercalated between systemic chemotherapy treatment in 29 (82.9%) patients and delivered at the end of chemotherapy treatment in 6 (17.1%) patients. The main clinical characteristics of patients who received HD‐MTX and those who did not were similar (Table S1). The median administration of IT prophylaxis was four courses (range, 1–6), and the majority of patients (*n* = 30, 88.2%) received methotrexate (MTX) combined with cytarabine. The doses of IT MTX and IT cytarabine were 10 and 50 mg, respectively. The median administration of HD‐MTX was four courses (range, 1–6) with a median dose of 3 g/m^2^ (range, 1.5–3.5 g/m^2^).

**TABLE 2:** First‐line treatment.

Treatment	All, *n* (%)
Numbers	135
Surgery	13 (9.6)
CT regimens	
CHOP	115 (85.2)
EPOCH	11 (8.1)
CHOEP	5 (3.7)
Hyper CAVD	4 (3.0)
Consolidative breast RT	61 (45.2)
CNS prophylaxis	69 (51.1)
IT only	34 (25.2)
HD‐MTX ± IT	35 (25.9)

Abbreviations: CHOEP, cyclophosphamide, doxorubicin, vincristine, prednisone, etoposide; CHOP, cyclophosphamide, doxorubicin, vincristine, prednisone; CNS, central nervous system; CT, chemotherapy; EPOCH, etoposide, cyclophosphamide, doxorubicin, vincristine, prednisone; HD‐MTX, high‐dose methotrexate; Hyper CVAD, hyper‐fractionated cyclophosphamide, vincristine, doxorubicin, dexamethasone; IT intrathecal; RT, radiotherapy.

### 3.3 Survival outcomes

**FIGURE 1:**
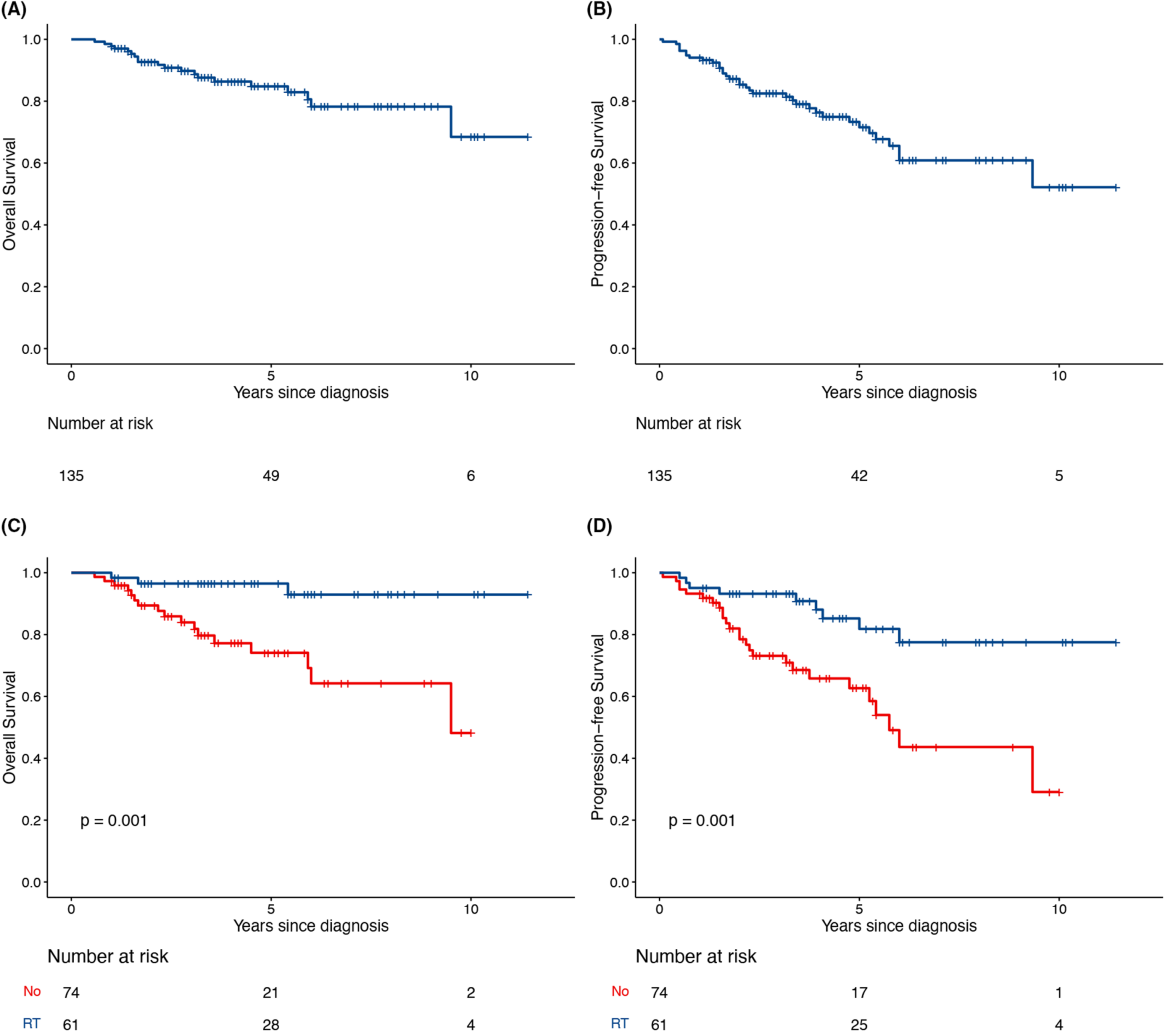
Survival outcomes and impact of treatment on PB‐DLBCL. (A) OS and (B) PFS. (C, D) Consolidative breast RT was associated with improvements in both OS and PFS. OS, overall survival; PB‐DLBCL, primary breast diffuse large B‐cell lymphoma; PFS, progression‐free survival; RT, radiotherapy.

With a median follow‐up of 4.2 years (95% confidence interval [CI], 3.2–5.2), the 5‐year PFS and OS rates were 71.6% (95% CI, 62.8–81.5%) and 84.8% (95% CI, 78.0–92.2%), respectively (Figure 1A,B).

Univariate analysis of risk factors for PFS and OS was carried out (Table S2). On multivariate analyses (Table 3), consolidative breast RT significantly improved both PFS (hazard ratio [HR], 0.293; 95% CI, 0.135–0.633; *p* = 0.002) and OS (HR, 0.185; 95% CI, 0.054–0.634, *p* = 0.007; Figure 1C,D). SM‐IPI was an independent prognostic factor for both PFS and OS.

**TABLE 3:** Multivariate analyses of OS and PFS for patients with PB‐DLBCL.

Characteristic	OS		PFS	
	HR (95% CI)	*p* value	HR (95% CI)	*p* value
SM‐IPI	2.854 (1.149–7.091)	0.024	2.112 (1.036–4.304)	0.040
RT	0.185 (0.054–0.634)	0.007	0.293 (0.135–0.633)	0.002
HD‐MTX	0.176 (0.023–1.321)	0.091	0.352 (0.123–1.005)	0.051

Abbreviations: CI, confidence interval; HD‐MTX, high‐dose methotrexate; HR, hazard ratio; OS, overall survival; PB‐DLBCL, primary breast diffuse large B‐cell lymphoma; PFS, progression‐free survival; SM‐IPI, stage modified International Prognostic Index, RT, radiotherapy.

### 3.4 Patterns of relapse

**FIGURE 2:**
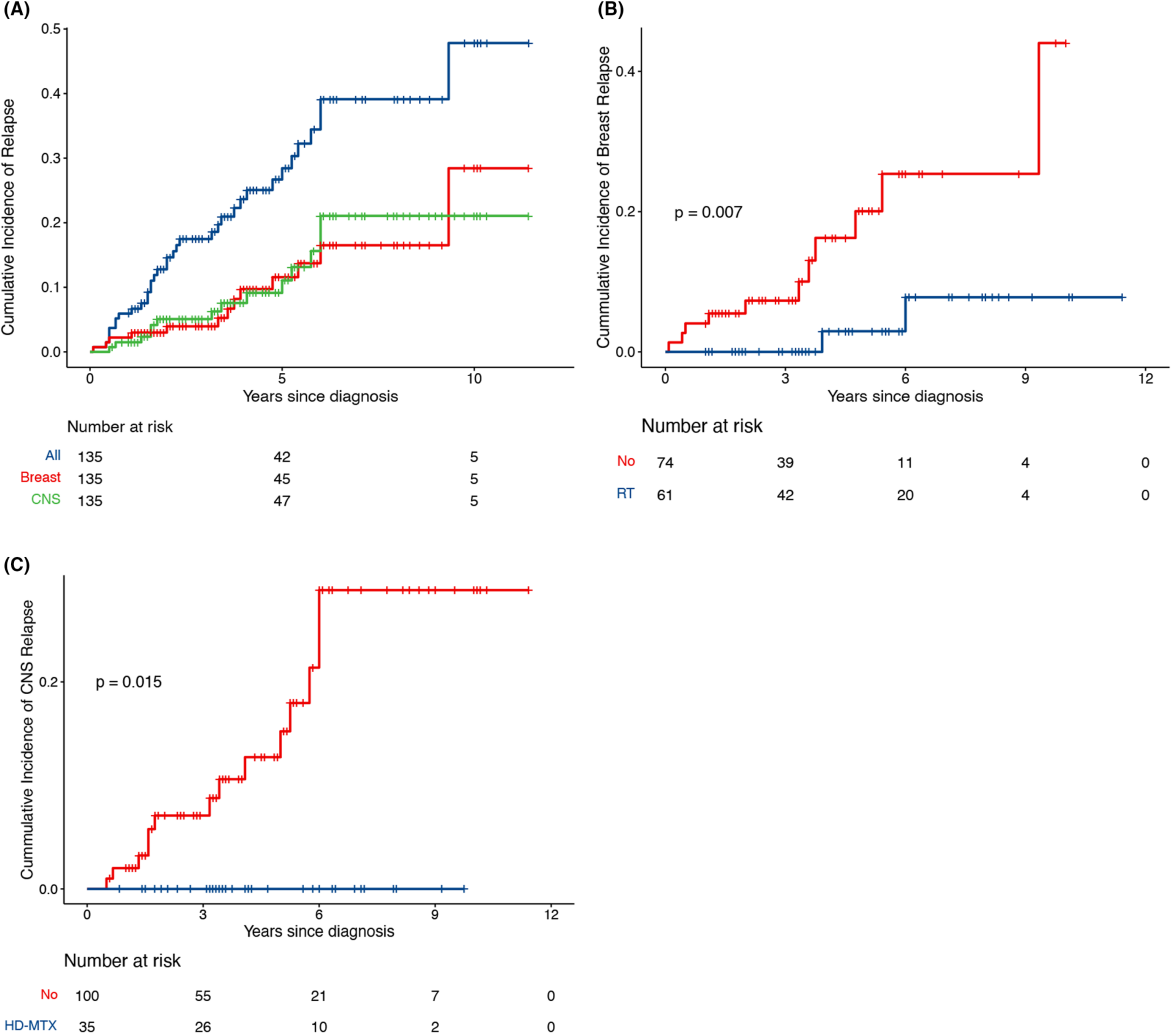
Cumulative incidence of relapse. (A) Cumulative relapse of all, breast and CNS, relapses. (B) Consolidative breast RT significantly reduced breast relapse risk. (C) HD‐MTX significantly reduced the risk of CNS relapse. CNS, central nervous system; HD‐MTX, high‐dose methotrexate; RT, radiotherapy.

Thirty‐five patients experienced disease progression or relapse at a median time of 24 months (range, 0.1–9.3 years), of which seven patients (20.0%) relapsed 5 years after treatment completion. The 5‐year cumulative incidence of relapses for all patients was 28.4% (Figure 2A). Extranodal relapse, with or without nodal disease, was reported in 32 patients (91.4%). The CNS and breast were the most common sites of relapse, while relapse was also observed in other extranodal and nodal sites. The details regarding relapse sites are shown in Table 4.

**TABLE 4:** Sites of lymphoma at relapse in 35 patients with PB‐DLBCL.

Relapse sites	All, *n* (%)
Numbers	35 (100)
Extranodal with or without nodal relapse	32 (91.4)
CNS relapse	14
Parenchyma	12
Leptomeninges	2
Breast relapse	12
Ipsilateral breast	5
Contralateral breast	7
Extranodal sites other than CNS or breast	6
Skin and/or soft tissue	3
Bone	1
Bone marrow	1
Liver	1
Nodal relapse only	3 (8.6)
Regional nodal relapse	1
Distance nodal relapse	2

Abbreviations: CNS, central nervous system; PB‐DLBCL, primary breast diffuse large B‐cell lymphoma.

Twelve patients experienced breast relapse, including 5 (41.7%) with relapse in the ipsilateral breast and 7 (58.3%) with relapse in the contralateral breast. The 5‐year cumulative incidence of breast relapse was 11.6%. Among the 61 patients who received RT, no patients relapsed in the radiation field, while two patients relapsed in the contralateral breast. Consolidative breast RT significantly reduced the cumulative incidence of breast relapses (5‐year risk, 2.9% vs. 20.1%, *p* = 0.007, Figure 2B).

CNS relapse occurred in 14 patients, of which 12 (85.7%) patients had isolated CNS relapses and 2 (14.3%) patients had concomitant CNS‐systemic relapses. Most CNS relapses occurred in the parenchyma (85.7%). The median time to CNS relapse was 39.5 months (range 6–72 months). The 5‐year cumulative incidence of CNS relapse was 11.1%. There is no significant difference in the risk of CNS relapse between patients who receive CNS prophylaxis (HD‐MTX and IT) and those who did not receive any CNS prophylaxis (*p* = 0.23, Figure S1). However, in terms of prophylactic routes, HD‐MTX significantly reduced the risk of CNS relapse compared to IT or no prophylaxis (5‐year risk, 0% HD‐MTX vs. 19.6% IT vs. 12.7% no prophylaxis, *p* = 0.048; Figure S2). In the subgroup of patients who received CNS prophylaxis, HD‐MTX significantly reduced CNS relapse risk compared to IT prophylaxis (*p* = 0.013). When analyzed separately, the CNS relapse rate was lower in patients who received HD‐MTX than in patients who did not receive HD‐MTX, with a 5‐year cumulative incidence of 0% and 15.2% (*p* = 0.015; Figure 2C).

Patients were treated with several treatment regimens after relapse. Twenty‐nine patients received salvage chemotherapy. Two patients were treated with high‐dose chemotherapy followed by autologous stem cell transplant. Three patients with isolated CNS relapse received whole‐brain RT after chemotherapy. Two patients received palliative care. Survival for patients after relapse is poor with a 1‐year OS rate of 56.6% (95% CI, 41.6%–76.9%).

### 3.5 Genomic mutation profiles

**FIGURE 3:**
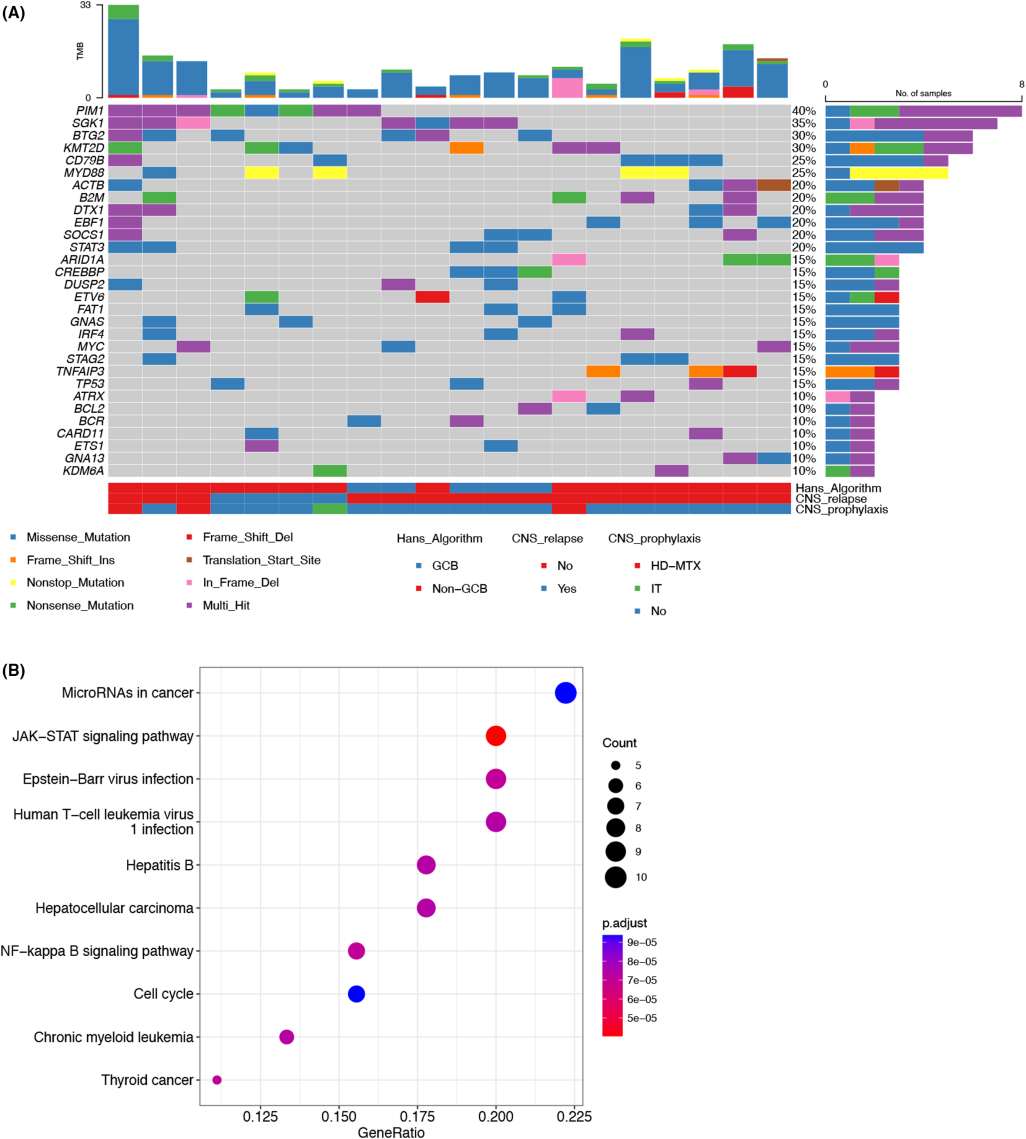
Genetic features in 20 patients with PB‐DLBCL. (A) The top 30 genes recurrently mutated in PB‐DLBCL. (B) Potential oncogenic pathways affected by exonic mutations in PB‐DLBCL. PB‐DLBCL, primary breast diffuse large B‐cell lymphoma.

To uncover the potential mechanisms involved in the pathogenesis of PB‐DLBCL, we performed targeted sequencing using a leukemia‐ and lymphoma‐related gene panel (Table S3) of 20 patients tumor samples from two institutes. The clinical characteristics of the 20 patients are summarized in Table S4. Among them, three received HD‐MTX and one received IT prophylaxis. A total of 460 exonic mutation events in 175 genes were identified. The most frequently mutated genes (≥15%) included *PIM1*, *SGK1*, *BTG2*, *KMT2D*, *CD79B*, *MYD88*, *ACTB, B2M*, *DTX1*, *EBF1*, *SOCS1*, *STAT3*, *ARID1A*, *CREBBP*, *DUSP2*, *ETV6*, *FAT1*, *GNAS*, *IRF4*, *MYC*, *STAG2*, *TNFAIP3*, and *TP53*. For *MYD88* mutation variants, p.L265p and p.X147R variants were identified. The spectrum of the top 30 genes recurrently mutated in PB‐DLBCL is presented in Figure 3A.

Interestingly, many of these recurrently mutated genes were associated with MCD‐enriched genes, including *PIM1* (40%), *CD79B* (25%), *MYD88* (25%), and *ETV6* (15%). KEGG pathway analysis revealed that many of the mutated genes are involved in cancer, signal transduction, infectious disease, and immune system pathways. Crucial signal transduction pathways included the JAK‐STAT, HBV infection, and NF‐kappa B signaling pathways (Figure 3B).

Four of the 20 patients experienced CNS relapse. Notably, all these four patients presented with *MYD88* and/or *CD79B* mutation and did not receive HD‐MTX as part of frontline treatment. In addition, three patients who presented with *MYD88* and/or *CD79B* mutation and received HD‐MTX did not experience CNS relapse.

## 4 DISCUSSION

To our knowledge, we present the largest report of PB‐DLBCL treatment and outcomes in the rituximab era. Based on a large continuous database of DLBCL, we found that PB‐DLBCL accounted for 2.7% of all DLBCLs in Chinese patients. Our results showed that rituximab‐containing immunochemotherapy produced an excellent response in patients with PB‐DLBCL; however, frequent relapse in extranodal sites, especially in the CNS and breast, remains the major treatment problem in the modern era.

For PB‐DLBCL, studies have revealed that consolidative breast RT improved outcomes and local control in PB‐DLBCL patients in the pre‐rituximab era.^5^, ^15^, ^19^, ^25^ However, the benefit of consolidative breast RT is still debated in the rituximab era. Consistent with previous studies,^5^, ^10^, ^17^, ^26^ breast failure was frequently observed even in patients who received rituximab‐containing regimens in this study. We found that consolidative breast RT significantly improved PFS and OS and reduced relapse rates. Similarly, in a retrospective multicenter study of 108 patients with PD‐DLBCL,^10^ 66 of whom received rituximab‐containing regimens, RT was associated with significantly better local control and improved outcomes in the subgroup of patients treated with rituximab. Of note, among patients who experienced breast relapse, the contralateral breast was the site of relapse in 58.3%, and none of the patients who received RT experienced ipsilateral breast relapse. We theorize that prophylactic RT to the contralateral breast may reduce the risk of recurrence. Because of the retrospective nature of the study, these hypotheses should be regarded as only suggestive but worthy of further exploration.

Our study showed that relapse involving the CNS was a major cause of treatment failure for PB‐DLBCL patients, with a 5‐year cumulative CNS relapse incidence of 11.1%. Previous studies reported that CNS relapse occurred in 5%–17% of patients with PB‐DLBCL, and breast involvement is generally considered a high‐risk factor for CNS relapse.^5^, ^10^, ^15^, ^17^, ^27^, ^28^ Therefore, CNS prophylaxis is recommended for PB‐DLBCL by some guidelines.^28^ However, several retrospective series showed that IT prophylaxis did not decrease CNS relapse risk in patients with PB‐DLBCL.^10^, ^17^ In a previous phase 2 study, although all patients received standard R‐CHOP and prophylaxis IT‐MTX, a high CNS relapse rate (12.5% at 2 years) was observed.^20^ In this study, 51.1% of patients received CNS prophylaxis, including 34 patients who received IT prophylaxis. However, we did not find a benefit of IT prophylaxis in PB‐DLBCL. These results suggest limited CNS prophylaxis efficacy of IT prophylaxis in patients with PB‐DLBCL and require a more effective prophylactic strategy. As most CNS relapses occur in the parenchyma, HD‐MTX has been considered a potentially better option.^10^, ^29^ Similarly, we also observed that a high proportion of CNS relapses occurred in the parenchyma. Our study showed that the addition of HD‐MTX significantly reduced the risk of CNS relapse in patients with PB‐DLBCL. Our results are in line with a recent study examining the impact of HD‐MTX on high‐risk DLBCL, in which breast involvement was considered high risk, which found that the addition of HD‐MTX was an independent factor in the prevention of CNS relapse.^30^ Due to the retrospective nature of this study, we did not analyze prophylaxis‐related toxicities. As a more toxic prophylactic approach, HD‐MTX should be administered in suitable patients.

The cause of distinct relapse patterns may support underlying biologic differences between PB‐DLBCL and common DLBCL. We observed frequently recurrent mutations in *MYD88* (25%) and *CD79B* (25%) in PB‐DLBCL, which was consistent with that observed in previous studies.^31^, ^32^, ^33^ Recurrent mutations in *MYD88* and *CD79B* have been reported to frequently occur in primary extranodal lymphoma, especially primary CNS lymphoma and primary testicular DLBCL.^34^, ^35^ Interestingly, we found frequent mutations in *MYD88/CD79B* in patients who developed CNS relapse. These mutations may represent a specific phenotype of aggressive DLBCL with a high risk of CNS relapse. Furthermore, we found that patients presenting with *MYD88/CD79B* mutations who received HD‐MTX as part of first‐line treatment did not experience CNS relapse. These results suggest the need for prophylactic HD‐MTX in patients with PB‐DLBCL, especially in patients with *MYD88/CD79B* mutations. Detection of the *MYD88/CD79B* mutation status may be considered routine management in patients with PB‐DLBCL.

Our study has several limitations. The major limitation is its retrospective nature. Only a small number of patients were included, and there was heterogeneity in the included patients treated at different institutions. Future studies are needed to determine whether *MYD88/CD79B* mutations are ultimately predictive of CNS relapse. Given the rarity of this disease and that randomized trials are virtually impossible, our results may be useful in clinical management decision‐making and may guide future study designs.

## 5 CONCLUSIONS

Our study provided a comprehensive summary of the clinical and genetic features of PB‐DLBCL in the rituximab era. Continuous extranodal relapse remains the main pattern of treatment failure in patients with PB‐DLBCL, especially breast and CNS relapse. We found that consolidative breast RT decreased the breast relapse risk and improved outcomes. Effective prophylaxis against CNS relapse can be provided with HD‐MTX.

## AUTHOR CONTRIBUTIONS

**Huawei Weng:** Formal analysis (equal); writing – original draft (lead). **Prem Raj Shrestha:** Formal analysis (equal); writing – original draft (equal). **Huangming Hong:** Formal analysis (equal); writing – original draft (equal). **Zegeng Chen:** Investigation (equal). **Le Yu:** Investigation (equal). **Yuyi Yao:** Investigation (equal). **Zhihui Zhang:** Resources (equal). **Liqun Zou:** Resources (equal). **Bo Zhu:** Resources (equal). **Hui Zhou:** Resources (equal). **Xianling Liu:** Resources (equal). **Yao Liu:** Resources (equal). **Hongqiang Guo:** Resources (equal). **He Huang:** Conceptualization (equal); project administration (equal); writing – review and editing (equal). **Tongyu Lin:** Conceptualization (equal); project administration (equal); writing – review and editing (equal).

## FUNDING INFORMATION

This study was supported by the National Natural Science Foundation of China (grant numbers 82003196 and 82270198), the Regional Innovation Cooperation Project of Science and Technology Department of Sichuan Province (grant number 2021YFQ0037), and Outstanding Young Scientific and Technological Talents Fund of Sichuan Province (grant number 2022JDJQ0059).

## CONFLICT OF INTEREST STATEMENT

The authors have no conflict of interest.

## ETHICS STATEMENT

This study was approved by the Institutional Review Board of Sun Yat‐sen University Cancer Center (Guangzhou, China, no. B2021‐470‐01). Written informed consent was obtained from each patient.

## Supporting information


Table S1.

## Data Availability

The data that support the findings of this study are available from the corresponding author upon reasonable request. The data are not publicly available due to privacy restrictions.
