# Supplementary material for: Primary breast diffuse large B‐cell lymphoma in the rituximab era: A retrospective study of the Chinese Southwest Oncology Group
Source: Cancer Med. 2023 Nov 23;12(23):21188–98. doi: 10.1002/cam4.6686 (PMC10726850; doi:10.1002/cam4.6686)

**Supporting Information**

***“Primary breast diffuse large B-cell lymphoma in the rituximab era: A retrospective study of the Chinese Southwest Oncology Group (CSWOG)”***

**List of Supporting Information**

***Supplementary Tables***

Table S1. Baseline characteristics by HD-MTX

Table S2. Univariate analyses of PFS and OS for PB-DLBCL

Table S3. Leukemia- and lymphoma-related genes panel for target-capture sequencing

Table S4. Clinical characteristics in 20 patients with PB-DLBCL

***Supplementary Figures***

Figure S1. Cumulative incidence of CNS relapse risk in patients received CNS prophylaxis or no prophylaxis

Figure S2. Cumulative incidence of CNS relapse risk by prophylactic strategy

Table S1. Baseline characteristics by HD-MTX

| **Characteristic** | **No HD-MTX (n=100)** | **HD-MTX (n=35)** | ***P*** |
| --- | --- | --- | --- |
| Age > 60 | 27 (27.0%) | 5 (14.3%) | 0.128 |
| B symptoms | 7 (7.0%) | 1 (2.9%) | 0.680 |
| Bulky disease (>7 cm) | 9 (9.0%) | 3 (8.6%) | 0.622 |
| Stage IIE | 51 (51.0%) | 19 (54.3%) | 0.738 |
| Elevated LDH | 25 (25.0%) | 7 (20.0%) | 0.549 |
| SM-IPI (2-3) | 26 (26.0%) | 7 (20.0%) | 0.477 |
| Non-GCB | 66 (66.0%) | 26 (74.3%) | 0.365 |
| *Abbreviations: LDH lactate dehydrogenase; SM-IPI, stage modified* *International Prognostic Index; GCB, germinal center B-cell-like; HD-MTX high-dose methotrexate* | | | |

Table S2. Univariate analyses of PFS and OS for PB-DLBCL

| **Characteristic** | **PFS** | | **OS** | |
| --- | --- | --- | --- | --- |
|  | **HR (95% CI)** | ***P value*** | **HR (95% CI)** | ***P value*** |
| Bulky disease | 1.305(0.398-4.277) | 0.661 | 2.748(0.797-9.476) | 0.109 |
| B symptoms | 0.640(0.152-2.694) | 0.543 | 1.207(0.276-5.287) | 0.803 |
| Bilateral involvement | 0.723(0.171-3.050) | 0.659 | 1.419(0.322-6.258) | 0.644 |
| SM-IPI (2-3 vs 0-1) | 2.071(1.023-4.190) | 0.043 | 3.347(1.358-8.248) | 0.009 |
| Cell of origin (Non-GCB) | 1.781(0.774-4.097) | 0.174 | 1.320(0.476-3.657) | 0.593 |
| Dual expression | 1.689(0.867-3.291) | 0.123 | 2.682(1.065-6.751) | 0.036 |
| RT | 0.303(0.141-0.651) | 0.001 | 0.170(0.050-0.582) | 0.001 |
| HD-MTX | 0.310(0.109-0.879) | 0.028 | 0.137(0.018-1.028) | 0.053 |
| IT | 1.658(0.810-3.392) | 0.167 | 1.499(0.576-3.904) | 0.407 |
| *Abbreviations: PFS, progression-free survival; OS, overall survival; HR, hazard ratio; CI, confidence interval; SM-IPI, stage modified International Prognostic Index; RT, radiotherapy; HD-MTX, high dose-methotrexate; IT intrathecal; PB-DLBCL, primary breast diffuse large B-cell lymphoma.* | | | | |

Table S3. Leukemia- and lymphoma-related genes panel for target-capture sequencing

| *ABCB1* | *CREBBP* | *LAMP1* | *PTPN6* | *BRCA2* | *ERCC3* | *NF1* | *SLC34A2* | *PBRM1* |
| --- | --- | --- | --- | --- | --- | --- | --- | --- |
| *ABCB4* | *CRLF2* | *LEF1* | *PTPRD* | *BRD4* | *ERCC4* | *NF2* | *SMAD2* | *PC* |
| *ABCC2* | *CSF1R* | *LMO1* | *PTPRO* | *BRIP1* | *ERCC5* | *NFKB1* | *SMAD4* | *SMARCA4* |
| *ABL1* | *CSF3R* | *LMO2* | *RAB27A* | *BTG2* | *GNAS* | *NFKB2* | *SMAD7* | *SMARCB1* |
| *ABL2* | *CTCF* | *LYN* | *RAC3* | *BTK* | *GRIN2A* | *NFKBIA* | *TMPRSS2* | *SMC1A* |
| *ACTA1* | *CTLA4* | *LYST* | *SMO* | *BTLA* | *GSTM1* | *NFKBIE* | *TNFAIP3* | *SMC3* |
| *ACTB* | *CTNNB1* | *MAF* | *SOCS1* | *BUB1B* | *GSTM4* | *NKX2-1* | *TNFRSF11A* |  |
| *ADH1B* | *CUX1* | *MAFB* | *SOX1* | *CALR* | *GSTM5* | *NKX2-2* | *TNFRSF14* |  |
| *AIM1* | *CXCR4* | *MALT1* | *SOX14* | *CARD11* | *GSTP1* | *NKX2-4* | *TNFRSF17* |  |
| *AIP* | *CYLD* | *MAP2K1* | *SOX2* | *CBFB* | *GSTT1* | *NOTCH1* | *TNFRSF19* |  |
| *AKT1* | *CYP19A1* | *MAP2K2* | *SOX21* | *CBL* | *HBA1* | *NOTCH2* | *TOP1* |  |
| *AKT2* | *CYP2A13* | *MAP2K4* | *SPEN* | *CBLB* | *HBA2* | *NPM1* | *TOP2A* |  |
| *AKT3* | *CYP2A6* | *MAP3K1* | *SPOP* | *CCND1* | *HBB* | *NQO1* | *TP53* |  |
| *ALDH2* | *ETV4* | *MAP3K14* | *SRC* | *CCND2* | *HDAC1* | *NRAS* | *TP63* |  |
| *ALK* | *ETV6* | *MCL1* | *SRSF2* | *CCND3* | *HDAC2* | *NSD1* | *TP73* |  |
| *AP3B1* | *EWSR1* | *MDM2* | *STAG2* | *CCNE1* | *HDAC4* | *NT5C2* | *TPMT* |  |
| *APC* | *EZH2* | *MDM4* | *STAT3* | *CCT6B* | *HDAC7* | *NTRK1* | *TRAF2* |  |
| *AR* | *FANCA* | *MED12* | *STAT5A* | *CD22* | *HGF* | *NTRK3* | *TRAF3* |  |
| *ARHGAP26* | *FANCC* | *MEF2B* | *STAT5B* | *CD274* | *HNF1A* | *NUP98* | *TRAF5* |  |
| *ARID1A* | *FANCD2* | *MEN1* | *STAT6* | *CD28* | *HNF1B* | *P2RY8* | *TSC1* |  |
| *ARID1B* | *FANCE* | *MET* | *STIL* | *CD58* | *HRAS* | *PAG1* | *TSC2* |  |
| *ARID2* | *FANCF* | *MGMT* | *STK11* | *CYP2A7* | *ID3* | *PAK3* | *TSHR* |  |
| *ARID5B* | *FANCG* | *MITF* | *STMN1* | *CYP2B6* | *IDH1* | *RAD21* | *TTF1* |  |
| *ASXL1* | *FANCL* | *MLH1* | *STX11* | *CYP2C19* | *IDH2* | *RAD50* | *TUBB2A* |  |
| *ATM* | *FAS* | *MLH3* | *STXBP2* | *CYP2C9* | *IGF1R* | *RAD51* | *TUBB2B* |  |
| *ATR* | *FAT1* | *MPL* | *SUFU* | *CYP2D6* | *IKBKE* | *RAF1* | *TUBB3* |  |
| *ATRX* | *FBXO11* | *PDCD1* | *SUZ12* | *CYP3A4* | *IKZF1* | *RARA* | *TYMS* |  |
| *AURKA* | *FBXW7* | *PDCD1LG2* | *SYK* | *CYP3A5* | *IKZF2* | *RASGEF1A* | *U2AF1* |  |
| *AURKB* | *FGFR1* | *PDE11A* | *TAL1* | *DAXX* | *IKZF3* | *RB1* | *UGT1A1* |  |
| *AXIN1* | *FGFR2* | *PDGFRA* | *TBL1XR1* | *DDR2* | *IL7R* | *RECQL4* | *UNC13D* |  |
| *AXL* | *FGFR3* | *PDGFRB* | *TCF3* | *DDX3X* | *INPP4B* | *RELN* | *VEGFA* |  |
| *B2M* | *FGFR4* | *PDK1* | *TCL1A* | *DHFR* | *INPP5D* | *RET* | *VHL* |  |
| *BAP1* | *FH* | *PGR* | *TEK* | *DICER1* | *IRF1* | *RHOA* | *WHSC1* |  |
| *BARD1* | *FIP1L1* | *PHF6* | *TEKT4* | *DNM2* | *IRF4* | *RICTOR* | *WT1* |  |
| *CD83* | *FLCN* | *PHOX2B* | *TERT* | *DNMT3A* | *IRF8* | *RNF43* | *XIAP* |  |
| *CDA* | *FLT1* | *PIK3CA* | *TET2* | *DNMT3B* | *JAK1* | *ROS1* | *XPC* |  |
| *CDC73* | *FLT3* | *PIK3CD* | *TGFBR2* | *DOT1L* | *JAK2* | *RPTOR* | *XPO1* |  |
| *CDH1* | *FLT4* | *PIK3R1* | *TLE1* | *DPYD* | *JAK3* | *RRM1* | *XRCC1* |  |
| *CDK10* | *FOXO1* | *PIK3R2* | *TLE4* | *DTX1* | *JARID2* | *RUNX1* | *YAP1* |  |
| *CDK12* | *FOXO3* | *PIM1* | *BCL10* | *DUSP2* | *JUN* | *RUNX1T1* | *ZAP70* |  |
| *CDK4* | *GADD45B* | *PLCG2* | *BCL11B* | *EBF1* | *MRE11A* | *SBDS* | *ZRSR2* |  |
| *CDK6* | *GATA1* | *PML* | *BCL2* | *ECSIT* | *MSH2* | *SDC4* | *CD70* |  |
| *CDK8* | *GATA2* | *PMS1* | *BCL2L1* | *ECT2L* | *MSH3* | *SDHA* | *CD74* |  |
| *CDKN1B* | *GATA3* | *PMS2* | *BCL2L11* | *EED* | *MSH6* | *SDHAP1* | *CD79A* |  |
| *CDKN1C* | *GNA11* | *POLE* | *BCL2L2* | *EGFR* | *MTHFR* | *SDHAP2* | *CD79B* |  |
| *CDKN2A* | *GNA13* | *POT1* | *BCL6* | *EGR1* | *MTOR* | *SDHAP3* | *ERG* |  |
| *CDKN2B* | *GNAQ* | *PPP2R1A* | *BCL7A* | *EP300* | *MUTYH* | *SDHB* | *ESR1* |  |
| *CDKN2C* | *KIT* | *PRDM1* | *BCOR* | *EPCAM* | *MYC* | *SDHC* | *ETS1* |  |
| *CEBPA* | *KLHL6* | *PRF1* | *BCORL1* | *EPHA2* | *MYCL1/MYCL* | *SDHD* | *ETV1* |  |
| *CEP57* | *KLLN* | *PRKAR1A* | *BCR* | *EPHA3* | *MYCN* | *SERP2* | *KDM2B* |  |
| *CHD8* | *KMT2A (MLL)* | *PTCH1* | *BIRC3* | *ERBB2* | *MYD88* | *SETBP1* | *KDM5A* |  |
| *CHEK1* | *KMT2B* | *PTEN* | *BLM* | *ERBB3* | *MYH11* | *SETD2* | *KDM6A* |  |
| *CHEK2* | *KMT2C* | *PTPN11* | *BMPR1A* | *ERBB4* | *NAT1* | *SF3B1* | *KDR* |  |
| *CIITA* | *KMT2D* | *PTPN13* | *BRAF* | *ERCC1* | *NBN* | *SGK1* | *PALB2* |  |
| *CKS1B* | *KRAS* | *PTPN2* | *BRCA1* | *ERCC2* | *NCSTN* | *SH2D1A* | *PAX5* |  |

Table S4. Clinical characteristics in 20 patients with PB-DLBCL

| **No** | **Sex** | **Age** | **Cell of origin** | **CNS prophylaxis** | **CNS relapse** |
| --- | --- | --- | --- | --- | --- |
| 1 | female | 39 | GCB | No | No |
| 2 | female | 43 | Non-GCB | No | No |
| 3 | female | 58 | Non-GCB | No | No |
| 4 | female | 46 | GCB | No | No |
| 5 | female | 52 | Non-GCB | No | Yes |
| 6 | female | 41 | Non-GCB | No | No |
| 7 | female | 37 | Non-GCB | IT | Yes |
| 8 | female | 57 | GCB | No | No |
| 9 | female | 36 | Non-GCB | HD-MTX | No |
| 10 | female | 64 | Non-GCB | No | No |
| 11 | female | 55 | Non-GCB | No | No |
| 12 | female | 49 | Non-GCB | No | No |
| 13 | female | 68 | Non-GCB | No | No |
| 14 | female | 56 | Non-GCB | No | No |
| 15 | female | 39 | Non-GCB | HD-MTX | No |
| 16 | female | 61 | Non-GCB | No | Yes |
| 17 | female | 40 | Non-GCB | No | Yes |
| 18 | female | 34 | Non-GCB | HD-MTX | No |
| 19 | female | 52 | GCB | No | No |
| 20 | female | 46 | GCB | No | No |
| *Abbreviations: GCB, germinal center; CNS, central nervous system; IT, intrathecal; HD-MTX, high-dose methotrexate; PB-DLBCL, primary breast diffuse large B-cell lymphoma.* | | | | | |

Figure S1. Cumulative incidence of CNS relapse risk in patients received CNS prophylaxis or no prophylaxis

Figure S2. Cumulative incidence of CNS relapse risk by prophylactic strategy
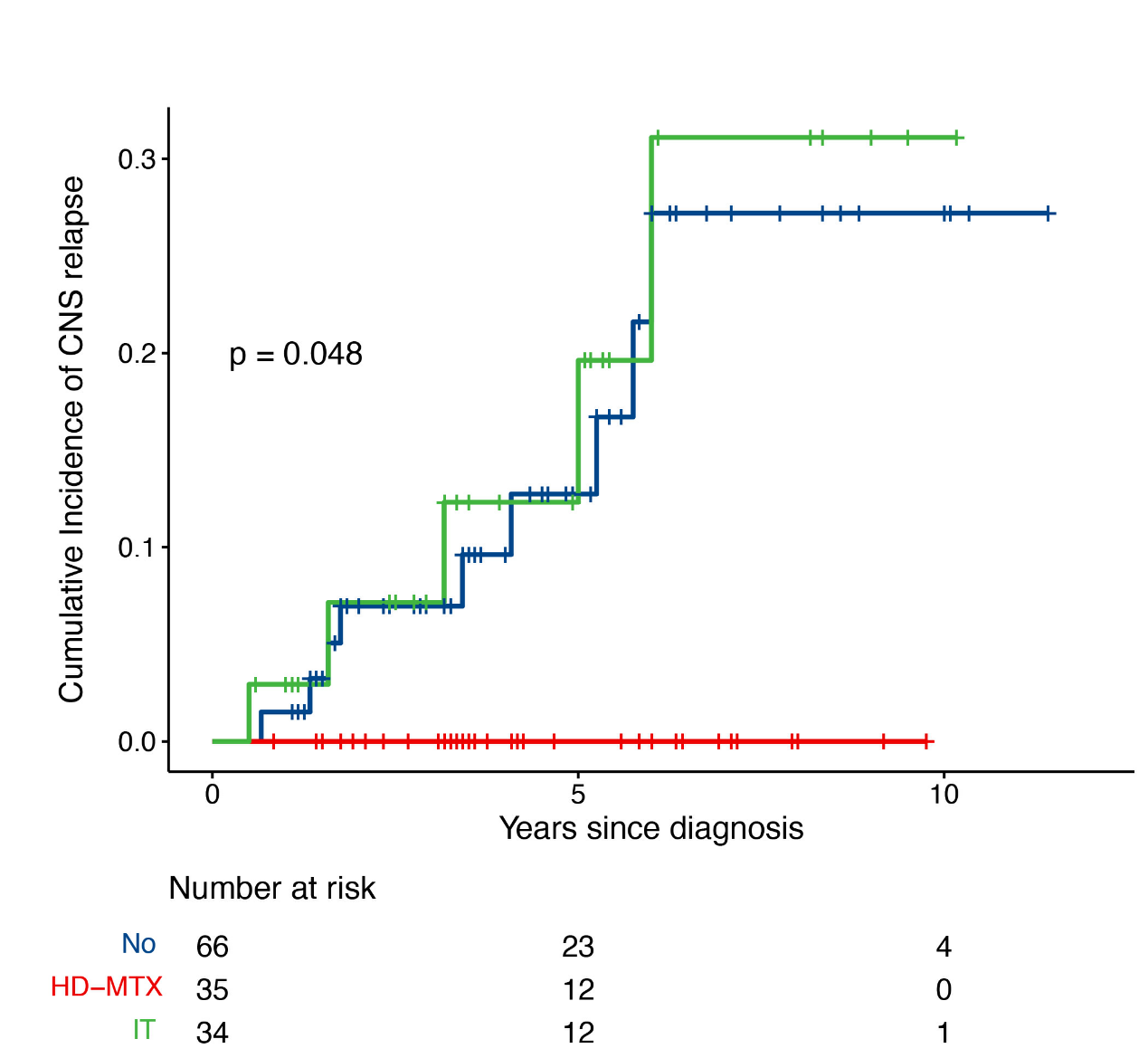

Supplement: Supplementary file 1 — Table S1. [file CAM4-12-21188-s001.docx]
